# Synthesis and Pharmacological Properties of Novel Esters Based on Monocyclic Terpenes and GABA

**DOI:** 10.3390/ph9020032

**Published:** 2016-06-13

**Authors:** Mariia Nesterkina, Iryna Kravchenko

**Affiliations:** Department of Pharmaceutical Chemistry, I.I. Mechnikov Odessa National University, Odessa 65026, Ukraine; kravchenko.pharm@gmail.com

**Keywords:** TRP channels, terpene esters, GABA, anticonvulsant activity, analgesic effect, anti-inflammatory action, co-administration

## Abstract

Novel esters of γ-aminobutyric acid (GABA) with monocyclic terpenes were synthesized via Steglich esterification and characterized by ^1^H-NMR, IR and mass spectral studies. Their anticonvulsant, analgesic and anti-inflammatory activities were evaluated by a PTZ-induced convulsion model, AITC-induced hyperalgesia and AITC-induced paw edema, respectively. All studied esters, as well as their parent terpenes, were found to produce antinociceptive effects in the AITC-induced model and attenuate acute pain more than the reference drug benzocaine after their topical application. GABA esters of l-menthol and thymol were also shown to exceed the reference drug ibuprofen in their ability to decrease the inflammatory state induced by intraplantar injection of the TRPA1 activator AITC. The present findings indicate that GABA esters of carvacrol and guaiacol are not a classical prodrug and possess their own pharmacological activity. Prolonged antiseizure action of the ester based on the amino acid and guaiacol (200 mg/kg) was revealed at 24 h after oral administration. Furthermore, orally co-administered gidazepam (1 mg/kg) and GABA esters of l-menthol, thymol and carvacrol produce synergistic seizure prevention effects.

## 1. Introduction

To date, significant progress had been made in our understanding of nociception and inflammatory reaction owing to the identification, cloning and characterization the transient receptor potential (TRP) family of cation channels. TRP channels are divided into seven subfamilies based on their amino acid sequence homology: TRPC, TRPV, TRPM, TRPN, TRPA, TRPP and TRPML, each of which can be activated by a number of phytochemicals [1,2]. For example, thymol and carvacrol can activate TRPV1 and TRPV3 ion channels; guaiacol may exert its antinociceptive effects via TRPV1; menthol was found to be a TRPM8 agonist [3]. Furthermore, the abovementioned monocyclic terpenes are active within the CNS and act as positive allosteric modulators of GABA_A_ receptors showing a variety of effects such as anticonvulsant, analgesic and nootropic actions [4,5].

γ-Aminobutyric acid (GABA) is well known as the main inhibitory neurotransmitter of the CNS, and its low levels are responsible for many pain states. Additionally, the experimental data and clinical studies clearly confirm an important role of GABA in the mechanism and treatment of epileptic seizures [6]. However, recent studies have provided strong evidences that GABA_B_ receptors are also localized in the periphery; surprising in this context is the finding of GABA localization at the terminal endings of corneal nociceptors [7]. Thus, the combination of TRP-channel modulators and compounds that bind to GABA receptors is expedient for development of novel drugs possessing a wide range of pharmacological activity.

Recently, we have reported the synthesis of GABA esters with l-menthol and thymol and the evaluation of their antiseizure and analgesic activity [8,9].The current study is a logical extension of our previous research aimed at synthesis and investigation of novel compounds with combined biological effects. In the present study, we investigated a range of pharmacological properties such as the anticonvulsant, analgesic and anti-inflammatory action of GABA esters with different terpenes.

## 2. Results and Discussion

### 2.1. Chemistry

**Scheme 1:**
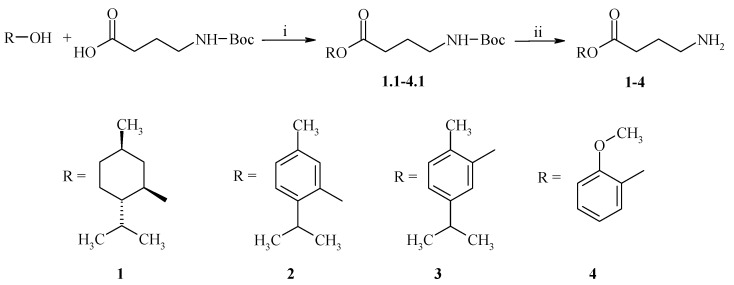
Synthetic pathway of compounds **1**–**4**. *Reagents and conditions*: (i) DMAP, CH_2_Cl_2_, rt, 10 min; DCC, 0 °C, 30 min; rt, 10 h; (ii) HCl, CH_3_COOH. All esters were prepared as hydrochlorides.

Synthesis of esters based on monocyclic terpenes and GABA (**1**–**4**) was carried out via Steglich esterification with *N*,*N*′-dicyclohexylcarbodiimide (DCC) and 4-dimethylaminopyridine (DMAP) as a catalyst in dichloromethane (DCM). The following terpenes were used for esters preparation: l-menthol (**5**), thymol (**6**), carvacrol (**7**), and guaiacol (**8**). Of these esters, compounds **1** and **2** had been prepared previously and are only shown herein for comparison. The condensation of 1.0 equivalent of an appropriate terpene with 1.05 equivalent of *tert*-butyloxycarbonyl (Boc)-protected GABA in the presence of DCC (1.1 equiv) and DMAP (catalytic amount) in dry DCM afforded the corresponding esters as yellow oily liquid (Scheme 1). The Boc group was removed with 1 M HCl in glacial acetic acid according to the literature procedure [10].

Synthesized esters of GABA were isolated as white (compound **3**) or brownish (compound **4**) solids and fully characterized by ^1^H-NMR, IR-spectroscopy and FAB-, ESI-mass spectrometry. The FAB-MS spectra of esters **3** and **4** display the protonated molecular ion peak [M + H]^+^ at *m*/*z* 236 and *m*/*z* 210, respectively. The HRMS (ESI-TOF) revealed an ion peak of compound **3** at m/z 236.1666 [M + H]^+^, thus suggesting a molecular formula C_14_H_22_NO_2_ (calc. 236.3300) as well as ion peak of ester **4** at *m*/*z* 210.2502 [M + H]^+^ corresponding to a molecular formula C_11_H_16_NO_3_ (calc. 210.2497). The IR spectra of esters **3** and **4** exhibit absorption bands of N–H bonds, C=O ester groups, aromatic C–H and alkyl C–H. The ^1^H-NMR spectral data contain resonance signals described by their chemical shift, integration and multiplicity that are in full agreement with the presented molecular formulas.

### 2.2. Pharmacology

#### 2.2.1. Antinociception Testing

TRP channels are broadly expressed in mammalian tissues; populations of non-neuronal cells within the skin express many different types of TPR channels making them a good therapeutic target for transdermal drug delivery [11]. Recently we have investigated the analgesic activity of esters **1**–**4** by pharmacological models of thermal and chemical stimuli (formalin and capsaicin) in mice after topical application in an ointment (2% *w*/*w*) [12]. All these pharmacological models are associated with activation of TRP channels: TRPV1, TRPV3 and TRPV4 contribute to acute noxious heat sensation; TRPA1 is targeted by formalin; capsaicin is TRPV1 agonist [13]. To expand our research and elucidate the influence of obtained esters on TRP channels, TRPA1 agonist allyl isothiocyanate (AITC) was used as a chemical stimulant with further examination of analgesic response in mice. For comparison, the initial monocycle terpenes—l-menthol (**5**), thymol (**6**), carvacrol (**7**) and guaiacol (**8**) have also been studied under the same experimental conditions. Benzocaine (BZC) was selected as reference drug because of the widespread use of this local anesthetic as a topical pain reliever and also based on ability of local anesthetics to activate TRPV1 and, to a lesser extent, TRPA1 channels [14]. The mice that have been treated with the base ointment (control group) exhibited the mean duration of AITC-induced nocifensive response of 71.3 ± 1.8 s (Table 1).

A statistically significant decrease of paw licking time was observed in mice treated with all studied compounds **1**–**8** (*p* < 0.01 *vs.* control mice). BZC, which served as a positive control in our experiment, also caused significant suppression of pain with the mean duration of licking time of 48.0 ± 2.0 s. It is noteworthy that all studied esters **1**–**4** and terpenes **5**–**8** better attenuated the pain reaction induced by subplantar AITC injection in comparison with positive control (BZC) as evidenced by licking time recorded for these compounds. The maximum analgesia was observed for *l*-menthol **5** (6.8 ± 1.2 s) and its GABA ester **1** (2.7 ± 0.3 s). When compared the licking time of mice treated with synthesized esters or terpenes, statistically significant difference was observed only between compounds **3** and **7** (*p* < 0.05). From these above results and due to the fact that GABA is localized to the terminal endings of corneal nociceptors, many of which also express TRPV1 [7], we may conclude that antinociceptive action of investigated esters was caused by contribution both the terpenes and GABA.

#### 2.2.2. Anti-Inflammatory Activity

**Figure 1:**
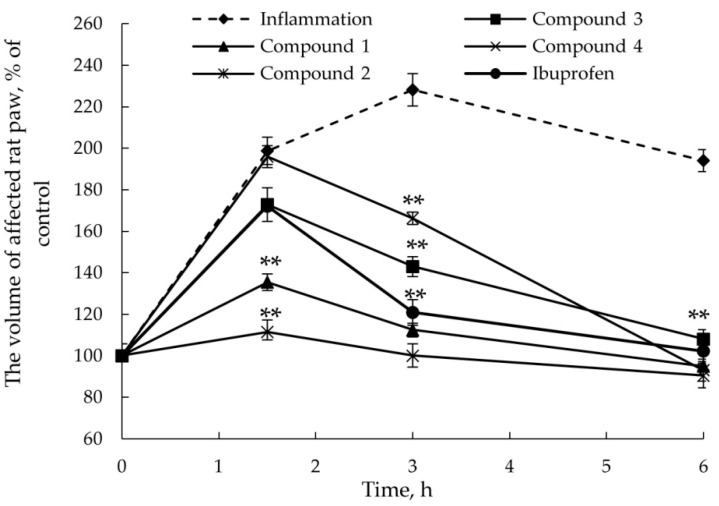
AITC-induced edema. Time dependence of the volume of affected rat paw after topical delivery (2% *w*/*w* ointment) of esters **1**–**4** and ibuprofen. ** *p* < 0.01 compared with vehicle-treated group. One-way analysis of variance (ANOVA) followed by Tukey’s *post hoc* comparison.

The activation of TRPA1 channels by AITC leads not only to the development of hyperalgesia but also results in inflammatory edema [15]. To examine the anti-inflammatory activity of GABA esters **1**–**4**, AITC was injected into the dorsal surface of the right rat paw at a dose selected by studying of the dose-response relationship − 100 μg/paw. Ibuprofen, which according to the literature data clearly inhibits inflammatory edema evoked by AITC, was selected in our research as a reference drug [16]. As shown in Figure 1, AITC-induced edema was maximal at 3 h and completely decreased to the basal level (105% ± 3.9%, data not shown) to 24 h after injection. As seen, after transdermal delivery (2% *w*/*w* ointment) of compounds **1**–**4** and ibuprofen the inflammation maximum was observed after 1.5 h following AITC injection. Our data indicate that the volume of affected rat paw used as a measure of edema (in percentage) reached the basal level (100%) at 6 h after AITC administration.

Topical application of all studied esters significantly reduced the inflammation level compared with vehicle-treated group of rats (inflammation curve). The highest anti-inflammatory action was demonstrated by compounds **1** and **2** which after topical application significantly decreased the level of AITC-induced edema to 136% and 112%, respectively; while the treatment with ibuprofen ointment reduced the volume of affected paw to 172%. Thus, esters **1** and **2** were found to exceed reference drug ibuprofen by their ability to decrease the inflammatory state induced by intraplantar injection of AITC, a TRPA1 activator.

#### 2.2.3. Anticonvulsant Action

Refractoriness to antiepileptic drugs is a major neurological problem, and efforts to identify alternative molecules are currently needed, especially for temporal lobe epilepsy which is the more common type of difficult to treat epileptic disorder [17]. Chemical seizure models are widely used in preclinical evaluation of drugs’ anticonvulsant properties [18]. Bearing in mind the fact that both carvacrol and guaiacol allosterically positively modulate GABA_A_ receptors [5] the convulsive behavior of experimental animals was induced by convulsant pentylenetetrazole (PTZ) which is GABA receptor agonist. Carvacrol and guaiacol esters of GABA were screened for its anticonvulsant potential trough PTZ test that represents a valid model for human generalized myoclonic seizures.

Previously, we have investigated the anticonvulsant activity of menthyl GABA ester **1** at a dose of 87–1350 mg/kg and shown that this compound exhibits antiseizure effect over the whole range of these doses. The dynamics of the action was extended studied for a dose of 175 mg/kg at 0.5–96 h after single oral administration; the maximum anticonvulsant effect was reached at 18 h with DCTC and DTE values 191% and 196%, accordingly [8]. Dose dependent anticonvulsant action was also detected for ester **2** over a range of doses 5–20 mg/kg. Investigation of time-response relationship (1–24 h) of compound **2** was carried out at a dose of 20 mg/kg. The findings have demonstrated the antiseizure effect throughout the whole time period with the following appropriate DCTC and DTE values: 263% and 256% at 1 h after administration; 181% and 183% at 24 h after administration [9].

**Figure 2:**
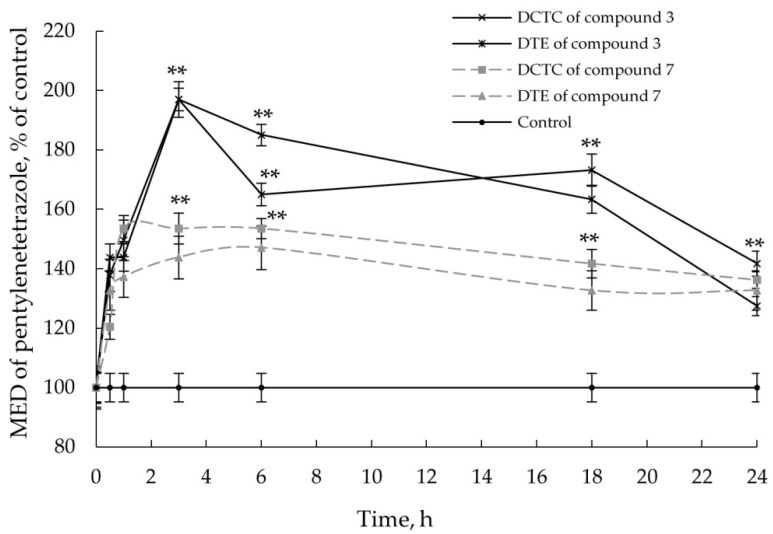
Anticonvulsant activity of compounds **3** and **7**; time-response relationship. Values are given as mean ± SEM, *n* = 5 mice; ** *p* < 0.01 compared with vehicle control. One-way analysis of variance (ANOVA) followed by Tukey’s *post hoc* comparison.

Based on the literature data [19], antiseizure effect was studied for carvacrol (200 mg/kg), guaiacol (200 mg/kg) and their esters with GABA (equimolar amounts) administered to mice orally in Tween 80/water emulsion, and Tween 80/water emulsion was used as a vehicle control. As illustrated in Figure 2, the maximum anticonvulsant action of ester **3** was observed at 3 h after its administration with DCTC and DTE values 197%.

For comparison, carvacrol was found to demonstrate maximal antiseizure activity between 1 h and 6 h after administration and show on the average the following data: 154% for DCTC and 144% for DTE. Considering the possible prolonged action of obtained esters, anticonvulsant evaluation was carried out throughout 24 h after single oral administration. According to our data ester treatment 24 h prior to PTZ intravenous administration produced antiseizure activity with DCTC and DTE values of 138% and 144%, accordingly indicating a low prolonged effect of compound **3**.

**Figure 3:**
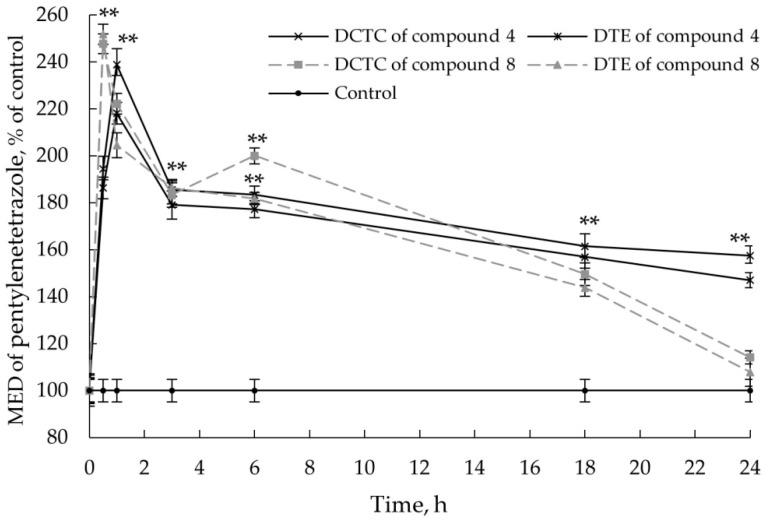
Anticonvulsant activity of compounds **4** and **8**; time-response relationship. Values are given as mean ± SEM, *n* = 5 mice; ** *p* < 0.01 compared with vehicle control. One-way analysis of variance (ANOVA) followed by Tukey’s *post hoc* comparison.

In the PTZ-induced convulsion model, both guaiacol and its ester **4** have shown similar antiseizure potency with a maximum at 1 h for ester **4** and 0.5 h for parent terpene after administration with appropriate DCTC and DTE values: 239% and 218% for compound **4**; 248% and 252% for compound **8** (Figure 3).

However, anticonvulsant action of ester **4** is retained at 24 h after administration proving that compound **4** possesses prolonged antiseizure effect: 157% and 147% for DCTC and DCTE, respectively. Bearing in mind that esters **3** and **4** demonstrate their effect at short time period (1–3 h), we may conclude that these compounds are not classical prodrugs and possess their own pharmacological activity.

#### 2.2.4. Co-Administration Effect of Gidazepam and GABA Esters **1**–**4**

**Figure 4:**
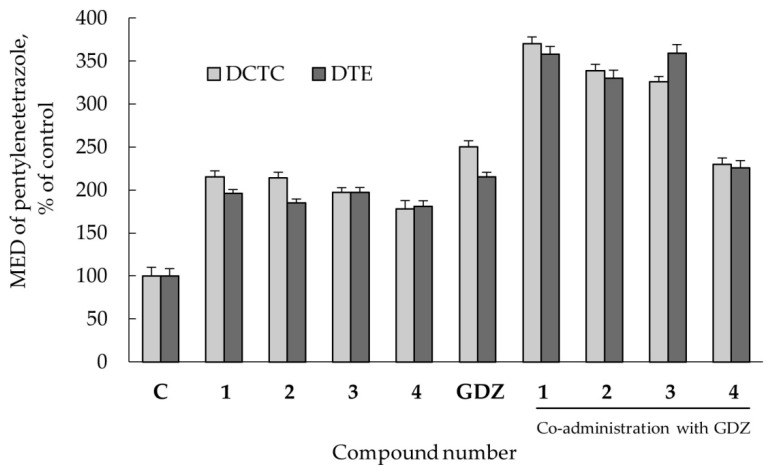
Anticonvulsant activity of compounds **1**–**4**, GDZ and mixtures of GDZ with **1**–**4** at 3 h after oral administration. For all groups *p* < 0.01 compared with control. One-way analysis of variance (ANOVA) followed by Tukey’s *post hoc* comparison.

Mice were distributed into 10 groups of five animals each, treated orally with gidazepam (GDZ, 1 mg/kg); ester **1** (175 mg/kg); ester **2** (20 mg/kg); esters **3** and **4** (200 mg/kg); mixtures of GDZ and esters **1**–**4**. As illustrated in Figure 4, all esters of GABA with monoterpenes display antiseizure effects in 3 h after oral administration as evidenced by increasing of DCTC and DTE values. GDZ (1 mg/kg) was found to protect against seizures with DCTC and DTE values of 250% and 215%, accordingly; whereas co-administration of GDZ and esters **5**–**7** was shown to increase anticonvulsant activity compared with each compound alone. It is noteworthy that GABA ester of guaiacol **4** combined with GDZ did not demonstrate a synergistic effect, which might be explained by structural differences of this compound in comparison with esters **1**–**3**.

Our experimental data demonstrate that orally co-administered gidazepam and GABA esters of monoterpenes (l-menthol, thymol and carvacrol) produce synergistic effect in seizures prevention suggesting that these esters are not acting *via* the benzodiazepine site; these results are in agreement with those obtained by electrophysiological investigation [20].

## 3. Materials and Methods

### 3.1. General

The following chemicals and drugs were used as obtained from their commercial suppliers: l-menthol, DMAP, GABA (Acros Organics, Geel, Belgium; Darmstadt, Germany), carvacrol, guaiacol, DCC, di-*tert*-butyl dicarbonate (TCI, Philadelphia, PA, USA), thymol (Sigma-Aldrich, Schnelldorf, Germany). Boc-protected GABA was not obtained commercially and has been synthesized according to the literature procedure [21]. Structures of the obtained compound were established by ^1^H-NMR spectroscopy on a AVANCE DRX 500 (500 MHz) instrument (Bruker, Davis, CA, USA) using DMSO-*d*_6_ and CDCl_3_ as solvents and TMS as an internal standard. FAB mass spectrum was obtained on a VG 70-70EQ mass spectrometer (VG Analytical Ltd., Manchester, UK) equipped with Xe ion gun (8 kV); the sample was mixed with *m*-nitrobenzyl-alcohol matrix. High-resolution mass spectrometry (HRMS) was performed on a 6530 Accurate Mass quadrupole time of flight (Q-TOF) spectrometer (Agilent, Santa Clara, CA, USA) using ESI (electrospray ionization) coupled to an Agilent 1260 Infinity HPLC system. IR spectra were measured with a Frontier FT-IR spectrometer (Perkin-Elmer, Hopkinton, MA, USA) using KBr pellets. The purity and identity of the compound were monitored by TLC on Merck-made (TLC Silica gel 60 F_254_) plates (Darmstadt, Germany).

### 3.2. General Procedure for the Synthesis of GABA Esters ***1***–***4***

To a stirred solution of terpene (0.5 g, 3.2 mmol) in CH_2_Cl_2_ (20 mL) at room temperature Boc-protected GABA (0.662 g, 3.26 mmol) and 4-dimethylaminopyridine (DMAP) (0.097 g, 0.794 mmol) were added. The reaction mixture was cooled to 0 °C, stirred for 10 min, and *N*,*N*′-dicyclohexylcarbodiimide (DCC) was added dropwise (0.727 g, 3.53 mmol). Stirring was continued for 30 min, then the flask was gradually warmed to the room temperature and the stirring continued for additional 10 h. Reaction completion was monitored by TLC. Reaction mixture was filtered, the filtrate was diluted to 100 mL and washed with 1 M aqueous HCl, 10% aqueous NaHCO_3_, and water. Deprotection of the N-Boc group was carried out using HCl/CH_3_COOH according to the literature procedure [10].

*(1R,2S,5R)-2-Isopropyl-5-methylcyclohexyl 4-[(tert-butoxycarbonyl)amino]butyrate* (**1.1**): Yellowish oil (80%). ^1^H-NMR (CDCl_3_, δ, ppm): 4.60*–*4.66 (td, 1H, H-1), 2.91 (t, 2H, γ-CH_2_), 2.19 (t, 2H, α-CH_2_), 1.82*–*1.90 (m, 1H, H-8), 1.71*–*1.78 (m, 2H, β-CH_2_), 1.53 (d, *J* = 12.10 Hz, 1H, H-6e), 1.42*–*1.46 (m, 1H, H-6a), 1.39 (s, 9H, Boc), 1.31*–*1.35 (m, 1H, H-5), 1.28*–*1.35 (m, 1H, H-2), 1.25*–*1.32 (m, 3H, H-4e + H-3e + H-3a), 1.16*–*1.24 (m, 1H, H-4a), 0.90 (d, *J* = 6.92 Hz, 3H, CH_3_*–*9), 0.81 (d, *J* = 6.53 Hz, 3H, CH_3_*–*7), 0.72 (d, *J* = 6.92 Hz, 3H, CH_3_*–*10). MS (FAB) *m*/*z*: 342 [M + H]^+^.

*2-Isopropyl-5-methylphenyl 4-[(tert-butoxycarbonyl)amino]butyrate* (**2.1**): Yellowish oil (75%). ^1^H-NMR (CDCl_3_, δ, ppm): 7.03 (d, *J* = 7.03 Hz, 1H, Ar-H), 6.49 (d, *J* = 7.28 Hz, 1H, Ar-H), 6.42 (s, 1H, Ar-H), 3.13*–*3.20 (m, 1H, CH), 2.91 (t, 2H, γ-CH_2_), 2.35 (t, 2H, α-CH_2_), 2.34 (s, 3H, CH_3_), 1.71*–*1.78 (m, 2H, β-CH_2_), 1.39 (s, 9H, Boc), 1.23 (d, *J* = 6.77 Hz, 6H, CH_3_). MS (FAB) *m*/*z*: 336 [M + H]^+^.

*5-Isopropyl-2-methylphenyl 4-[(tert-butoxycarbonyl)amino]butyrate* (**3.1**): Yellowish oil (75%). ^1^H-NMR (CDCl_3_, δ, ppm): 7.17 (d, *J* = 6.90 Hz, 1H, Ar-H), 7.06 (d, *J* = 6.73 Hz, 1H, Ar-H), 6.76 (s, 1H, Ar-H), 3.16 (t, 2H, γ-CH_2_), 2.82 (t, 2H, α-CH_2_), 2.37 (s, 3H, CH_3_), 2.27*–*2.33 (m, 1H, CH), 1.81*–*1.86 (m, 2H, β-CH_2_), 1.43 (s, 9H, Boc), 1.14 (d, *J* = 5.22 Hz, 6H, CH_3_). MS (FAB) *m*/*z*: 336 [M + H]^+^.

*2-Methoxylphenyl 4-[(tert-butoxycarbonyl)amino]butyrate* (**4.1**): Yellowish oil (77%). ^1^H-NMR (CDCl_3_, δ, ppm): 7.20 (t, 1H, Ar-H), 7.05 (t, 1H, Ar-H), 6.98 (d, *J* = 8.00 Hz, 1H, Ar-H), 6.95 (d, *J* = 8.01 Hz, 1H, Ar-H), 3.79 (s, 3H, CH_3_), 3.16 (t, 2H, γ-CH_2_), 2.82 (t, 2H, α-CH_2_), 1.79*–*1.86 (m, 2H, β-CH_2_), 1.43 (s, 9H, Boc). MS (FAB) *m*/*z*: 310 [M + H]^+^.

Compounds **1** and **2** were synthesized and characterized in our previous studies [8,9].

(*1R,2S,5R)-2-Isopropyl-5-methylcuclohexyl 4-aminobutyrate hydrochloride* (**1**): White solid (96%). IR ν_max_ (cm^−1^): 3021, 2957*–*2868, 1721, 1573, 1604. ^1^H-NMR (DMSO-*d*_6_, δ, ppm): 4.53*–*4.59 (td, 1H, H-1), 2.74 (t, 2H, α-CH_2_), 2.37 (m, 2H, β-CH_2_), 1.82 (d, *J* = 12.10 Hz, 1H, H-6e), 1.76 (t, 2H, γ-CH_2_), 1.59 (m, 2H, H-3e + H-4e ), 1.40 (m, 1H, H-3a), 1.27–1.32 (m, 1H, H-5), 0.96–1.01 (m, 1H, H-2), 0.87–0.93 (m, 1H, H-6a), 0.81–0.84 (m, 7H, CH_3_-9,10 + H-4a), 0.67 (d, 3H, J = 6.53, CH_3_-7). MS (FAB) *m*/*z*: 242 [M + H]^+^.

*2-Isopropyl-5-methylphenyl 4-aminobutyrate hydrochloride* (**2**): White solid (80%). IR ν_max_ (cm^−1^): 3430, 3330, 2929*–*2851, 1723, 1577, 1244. ^1^H-NMR (DMSO-*d*_6_, δ, ppm): 7.18 (d, *J* = 7.03 Hz, 1H, Ar-H), 6.99 (d, *J* = 7.28 Hz, 1H, Ar-H), 6.80 (s, 1H, Ar-H), 2.79*–*2.86 (m, 1H, CH), 2.29 (t, 2H, α-CH_2_), 2.21 (s, 3H, CH_3_), 1.87*–*1.92 (m, 2H, β-CH_2_), 1.74 (t, 2H, γ-CH_2_), 1.06 (d, *J* = 6.77 Hz, 6H, CH_3_). MS (FAB) *m*/*z*: 236 [M + H]^+^.

*5-Isopropyl-2-methylphenyl 4-aminobutyrate hydrochloride* (**3**): White solid (85%). IR ν_max_ (cm^−1^): 3429, 3219, 2957*–*2868, 1734, 1377, 1180, 826. ^1^H-NMR (DMSO-*d*_6_, δ, ppm): 7.18 (d, *J* = 6.87 Hz, 1H, Ar-H), 7.04 (d, *J* = 6.58 Hz, 1H, Ar-H), 6.92 (s, 1H, Ar-H), 2.76 (t, 2H, α-CH_2_), 2.29*–*2.38 (m, 1H, CH), 2.05 (s, 3H, CH_3_), 1.95 (t, 2H, γ-CH_2_), 1.76*–*1.79 (m, 2H, β-CH_2_), 1.16 (d, *J* = 5.22 Hz, 6H, CH_3_). MS (FAB) *m*/*z*: 236 [M + H]^+^. HRMS (ESI-TOF) calculated for C_14_H_22_NO_2_ [M + H]^+^ 236.3300, found 236.1666.

*2-Methoxyphenyl 4-aminobutyrate hydrochloride* (**4**): Brownish solid (80%). IR ν_max_ (cm^−1^): 3433, 3051, 2918, 1720, 1505, 1215, 771. ^1^H-NMR (DMSO-*d*_6_, δ, ppm): 7.20 (t, 1H, Ar-H), 7.06 (t, 1H, Ar-H), 7.03 (d, *J* = 8.01 Hz, 1H, Ar-H), 6.94 (d, *J* = 8.01 Hz, 1H, Ar-H), 3.79 (s, 3H, CH_3_), 2.77 (t, 2H, α-CH_2_), 2.33 (t, 2H, γ-CH_2_), 1.74*–*1.80 (m, 2H, β-CH_2_). MS (FAB) *m*/*z*: 210 [M + H]^+^. HRMS (ESI-TOF) calculated for C_11_H_16_NO_3_ [M + H]^+^ 210.2497, found 210.2502.

### 3.3. Animals

Anticonvulsant and analgesic activities of synthesized compound were studied using outbreed male white mice (18*–*22 g) as experimental animals. Investigation of the anti-inflammatory effect was carried out in male Wistar rats (150*–*180 g). All animals were kept under 12 h light regime and in a standard animal facility with free access to water and food, in compliance with the European Convention for the Protection of Vertebrate Animals Used for Experimental and Other Specific Purposes (Strasbourg, 1986) and the principles of the National Ukrainian Bioethics Congress (Kyiv, 2003). All the animals were purchased from Odessa National Medical University, Ukraine. The Animal Ethics Committee (agreement No. 29/2016) of Odessa National University (Ukraine) approved the study.

### 3.4. Drug Administration

Topical application of esters **1***–***4** and initial monoterpenes **5***–***8** were used in order to evaluate their analgesic and anti-inflammatory effect. The ointments (2% *w*/*w*) were prepared using a mixture of polyethylene glycol (PEG 1500), polyethylene oxide (PEO 400) and 1,2-propylene glycol in the ratio of 4:2:3 as a base. Antiseizure action was studied for carvacrol and guaiacol at a dose of 200 mg/kg and their esters with GABA (equimolar amounts); all compounds were administered to mice orally in Tween 80/water emulsion, and Tween 80/water emulsion was used as a vehicle control.

### 3.5. Antinociception Testing

The antinociceptive activity was evaluated by model of chemical stimulus with the use of the TRPA1 agonist allyl isothiocyanate (AITC). The method used was similar to that described in [22]. Following the adaptation to the experimental conditions, 20 μL of 0.5% (*w*/*w*) AITC solution was injected subcutaneously under the skin of the dorsal surface of the right hindpaw. The ointments were applied to the plantar surface of the right paw by gently rubbing 5 min before antinociceptive activity evaluation. Control animals received only a corresponding amount of the ointment base. AITC dose was selected based on our preliminary trials and from literature citations. Animals were observed individually for 10 min after AITC administration. The amount of time that mice spent licking the injected paw (reaction time in seconds) was recorded and considered as an index of pain.

### 3.6. AITC-Induced Acute Inflammatory Model

Anti-inflammatory activity of compounds **1***–***4** and reference drug ibuprofen was determined using an AITC-induced rat paw edema assay. Edema was induced by subplantar injection of 30 μL of AITC solution (100 μg/paw) in propylene glycol 30 min prior to ointment application. Edema was measured before (control data) and at 1.5, 3, 6 and 24 h after AITC injection by using a plethysmometer. The ointments were additionally applied by gently rubbing on the affected paw at each time point. Values of paw volume at each time point are expressed as a percentage relative to control (non-inflamed paw).

### 3.7. Pentylenetetrazole-Induced Convulsions in Mice

The anticonvulsant activity of tested compounds was evaluated by pentylenetetrazole model (PTZ), which includes the determining of pentylenetetrazole minimum effective doses (MED) inducing clonic-tonic convulsions (CTC) and tonic extension (TE) in test animals upon intravenous infusion of 1% aqueous solution into a tail vein. Doses of pentylenetetrazole for inducing clonic-tonic convulsions (DCTC) and tonic extension (DTE) were calculated relative to control. The anticonvulsant effect of compounds was estimated at certain time points (0.5, 1, 3, 6, 18 and 24 h) from the increase of pentylenetetrazole MED compared with a control group. MED in percent was calculated using the formula:

(1) $$\text{MED }\left(\%\right)\;=\;\frac{V}{m}\;\times 10^{4}$$

where MED—minimum effective dose of PTZ inducing DCTC or DTE; *V*—volume of PTZ solution, mL; *m*—animal weight, g.

### 3.8. Co-Administration Effect of Gidazepam and GABA Esters ***1**–**4***

Mice were distributed into 10 groups of five animals each, treated orally with gidazepam 1 mg/kg (GDZ); GABA ester of menthol 175 mg/kg (**1**); GABA ester of thymol 20 mg/kg (**2**); GABA ester of carvacrol 200 mg/kg (**3**); GABA ester of guaiacol 200 mg/kg (**4**); mixture of GDZ and **1**; mixture of GDZ and **2**; mixture of GDZ and **3**; mixture of GDZ and **4**. Doses of esters **1**–**4** were calculated in equimolar amounts with respect to monoterpenes based on our preliminary investigation and from literature citations [8,9,18]. The anticonvulsant activity of compounds **1**–**4** and GDZ as well as mixtures of GDZ with **1**–**4** was evaluated in model of acute generalized seizures as described above; pharmacological effect of compounds was estimated in 3 h.

### 3.9. Statistical Analysis

All results are expressed as mean ± standard error mean (SEM). One-way analysis of variance (ANOVA) was used to determine the statistical significance of the results followed by Tukey’s *post hoc* comparison. *p* < 0.05 was considered as significant.

## 4. Conclusions

We have synthesized novel esters of GABA with some monocyclic terpenes (l-menthol, thymol, carvacrol and guaiacol) and demonstrated their high analgesic and anti-inflammatory activities. Prolonged anticonvulsant action was found for ester **4**; compounds **1**–**3** additionally produce synergistic seizure prevention effects when co-administered with gidazepam. Thus, we showed the possibility of development of novel drugs possessing a wide range of pharmacological activity which are simultaneously able to modulate TRP-channels and bind to GABA receptors.

## Figures and Tables

**Table 1:** Analgesic activity of compounds **1**–**8** in the AITC test in mice (2% *w*/*w* ointment).

Compound	Reaction Time (in s)	Compound	Reaction Time (in s)
Control	71.3 ± 1.8	**4**	25.3 ± 1.5
Benzocaine	48.0 ± 2.0	**5**	6.8 ± 1.2
**1**	2.7 ± 0.3	**6**	25.3 ± 3.8
**2**	20.3 ± 5.8	**7**	34.5 ± 2.2
**3**	22.8 ± 4.3	**8**	20.7 ± 2.8

All values are expressed as mean ± SEM; *n* = 6 mice; for all groups *p* < 0.01 compared with control group. One-way analysis of variance (ANOVA) followed by Tukey’s *post hoc* comparison.
